# METTL14 promotes glomerular endothelial cell injury and diabetic nephropathy via m6A modification of α-klotho

**DOI:** 10.1186/s10020-021-00365-5

**Published:** 2021-09-09

**Authors:** Manna Li, Le Deng, Gaosi Xu

**Affiliations:** Department of Nephrology, The Second Affiliated Hospital to Nanchang University, No. 1, Minde Road, Donghu District, Nanchang, 330006 China

**Keywords:** Diabetic nephropathy, Glomerular endothelial cell injury, m6A, METTL14, α-Klotho

## Abstract

**Background:**

N6-Methyladenosine (m6A) modification has been implicated in many bioprocesses. However, its functions in diabetic nephropathy (DN) have not been determined. Here, we investigated the role of METTL14, a key component of the m6A methyltransferase complex, in DN.

**Methods:**

The expression of METTL14 was detected in DN patients and human renal glomerular endothelial cells (HRGECs). In vitro and in vivo experiments were performed to explore the functions of METTL14 on high glocse-induced HRGECs and renal injury of DN mice. We also investigated whether METTL14 works by regulating α-klotho expression through m6A modification.

**Results:**

METTL14 were highly expressed in kidneys of DN patients and high glocse-induced HRGECs both at the mRNA and protein level. Overexpression of METTL14 increased ROS, TNF-α and IL-6 levels and apoptosis in HRGECs. Conversely, METTL14 silence decreased the levels of ROS, TNF-α and IL-6 and cell apoptosis. We confirmed that METTL14 down-regulated α-klotho expression in an m6A-dependent manner. In addition, we also found that METTL14 aggravated renal injury and inflammation of db/db mice, which could partially rescued by α-klotho.

**Conclusion:**

Our data revealed that METTL14 plays a vital role in high glucose-induced glomerular endothelial cells and diabetic nephropathy through m6A modification of α-klotho.

## Background

Diabetic nephropathy (DN) is the most common microvascular complication in diabetes and a chronic progressive kidney disease which causes the end-stage renal disease (Vasanth Rao et al. 2019). The occurrence and development of DN may be caused by an interaction between inflammation, metabolism, and hemodynamics, which leads to increased glomerular injury and molecular modifications under hyperglycemia conditions (Warren et al. 2019; Rayego-Mateos et al. 2020). The pathogenesis of DN is complex, and the effect of existing treatment methods is limited. Therefore, further exploration of the molecular mechanisms of DN will help to find potential therapeutic targets and provide new treatment options.

N6-methyladenosine (m6A) modification is the most abundant and conservative reversible post-transcriptional modification in the mRNA of bacteria and eukaryotic cells (Wei et al. 2017; Bi et al. 2019). M6A is mediated by a methyltransferase complex, which contained the methyltransferase-like enzymes METTL3 and METTL14 (Liu et al. 2020, 2014). In contrast, m6A on RNA can be removed by demethylase such as FTO and ALKBH5 (Gao et al. 2019; Zou et al. 2016). The m6A modification regulates the splicing, transport, stability and translation efficiency of RNA, and further participates in the biological processes of metabolic diseases such as obesity and diabetes (Wu et al. 2020). Yang et al. found that in patients with type 2 diabetes, the m6A content was decreased. Interestingly, the mRNA expression levels of METTL3, METTL14, FTO, and WTAP were increased (Yang et al. 2019; Shen et al. 2015). METTL3/METT14 deleted mice developed hyperglycemia and hypoinsulinemia by regulating β-cell development and glycemic control (Wang et al. 2020). METT14 deficiency enhanced AKT signaling activation and decreased gluconeogenesis, playing a key role in β-cell survival, insulin secretion and glucose homeostasis (Liu et al. 2019). Similarly, in diabetic complications such as diabetic cataract (Yang et al. 2020), METTL3 was upregulated in the diabetic cataract tissue specimens and high glucose-induced lens epithelial cells. METTL3 knockdown promoted the proliferation and repressed the apoptosis of lens epithelial cells. Zha and his colleagues (2020) have found that METTL3 rescued cell viability in high-glucose treated retinal pigment epithelium cells by targeting miR-25-3p/PTEN/Akt signaling cascade in diabetic retinopathy. However, the role of m6A in the pathogenesis of DN is still unknown.

Klotho is identified as an anti-aging gene and is involved in human health and disease (Kuro 2019). The protein encoded by klotho gene has many biological effects, such as anti-inflammatory response, anti-oxidative stress, anti-apoptosis, and anti-fibrosis. In recent years, it has been reported that α-klotho plays a protective role in DN (Xiong and Zhou 2019). In our previous study, we also found that α-klotho prevented renal tubular and glomerular injury and attenuated diabetic nephropathy in diabetic mice (Kang and Xu 2016; Li et al. 2019a; Wang et al. 2019). It has become clear that epigenetic processes such as DNA methylation, histone modifications and non-coding RNA regulation are essentially involved in Klotho gene expression (Li et al. 2019a; Zhu et al. 2019; Han and Sun 1979). Chen et al. (2019) have shown that α-klotho mRNA is hypermethylated by METTL14, resulting in the α-klotho mRNA de-expression. In the current study, we investigated the functions of METTL14 on glomerular endothelial cell injury in vitro and diabetic nephropathy in vivo, and explored whether METTL14 works through mediating m6A modification of α-klotho.

## Materials and methods

### Clinical samples

This study was approved by the Ethics Committee of the Second Affiliated Hospital of Nanchang University. The renal samples of 20 DN patients were collected from the Second Affiliated Hospital of Nanchang University. Twenty control samples were obtained from normal adjacent tissues of renal carcinoma patients who underwent tumor nephrectomies without diabetes or other renal diseases.

### Animals

All animal experiments were approved by Animal Ethical Committee of Nanchang University. A total of 30 db/db mice were purchased from Model Animal Research Center of Nanjing University. After adaptive feeding for 1 week, db/db mice were randomly divided into five groups (n = 6): db/db group, db/db + rAAV group, db/db + rAAV-METTL14 group, db/db + rAAV-klotho group, and db/db + rAAV-METTL14 + rAAV-klotho group. Except db/db group, the other four groups were injected with recombinant adeno-associated virus (rAAV) control, rAAV mediated delivery of METTL14 (rAAV-METTL14), or/and rAAV mediated delivery of klotho (rAAV-klotho) respectively via tail vein. Six db/m mice were chosen as the normal control. After 8 weeks of injection, mice were sacrificed, and the blood and kidney tissues were collected. The 24-h urine protein, kidney weight (KW) and body weight (BW) were assayed and the kidney hypertrophy index (KHI) was calculated according to the formula: KHI = KW/BW.

### Histopathological analysis

For assessment of kidney injury, renal sections were stained with hematoxylin and eosin (H&E) and Masson. Briefly, renal tissues of each mouse were fixed in 4% paraformaldehyde, embedded in paraffin, and then sectioned at 4 μm thickness. H&E staining (Abcam, UK) was performed to detect general morphological changes and Masson staining (Sigma-Aldrich, USA) was assessed to examination of matrix deposition within the interstitium according to standard protocols.

### Cell culture

Human renal glomerular endothelial cells (HRGECs) purchased from ScienCell Research Laboratories were maintained in Endothelial Cell Medium (ECM, Carlsbad, CA, USA) containing 5.6 mmol/L glucose and 10% fetal bovine serum (FBS; Gibco). To induce disease model in vitro, HRGECs were exposed to 20 mmol/L d-glucose, named high glucose (HG) group. In addition, HRGECs were exposed to 5.6 mmol/L d-glucose, as normal glucose (NG) group, and to 5.6 mmol/L d-glucose + 14.4 mmol/L mannitol, as an osmotic pressure control, named high mannitol (HM) group.

### Plasmid construction and cell transfection

To construct expression plasmids of METTL14 and α-klotho, the sequences of wild-typed METTL14 or klotho were amplified respectively and cloned into the pcDNA 3.1 vector (Invitrogen, USA). The specific siRNAs against METTL14 were designed and synthesized by GenePharma (Shanghai, China). When HRGECs reached 80% confluence, they were transfected with plasmids or siRNAs using Lipofectamine (Invitrogen) according to the manufacturer’s instructions. The siRNA with the highest knockdown efficiency was selected for the further research.

### Cell counting kit-8 (CCK8) assay

Cell proliferation was tested by CCK8 (Beyotime, China). HRGECs with different treatment were cultured in a 96-well plate for 24 h, 48 h and 72 h respectively and then incubated with CCK-8 reagent. The proliferation was assessed via the absorbance at 450 nm using a microplate reader (Thermo Fisher Scientific).

### Terminal deoxynucleotidyl transferase-mediated dUTP nick end labeling (TUNEL) staining

The apotosis of HRGECs was examined by TUNEL assay kit (Beyotime, China) according to the manufacturer’s directions. DAPI was used to locate the nuclei of the cells. The number of TUNEL-positive cells was counted and the ratio of TUNEL-positive cells to total cells indicates the apoptosis index.

### Enzyme linked immunosorbent (ELISA) assay

The ROS (#DRE901Mu, ShangHaiLianShuo Biological Co., Ltd.), SOD (R&D Systems, #DYC3419-2) and MDA (#EU2577, Wuhan Fine Biotech Co., Ltd.) levels in mice renal tissues and α-klotho (R&D Systems, #AF1819), TNF-α (R&D Systems, #MTA00B) and IL-6 (Sigma, #RAB0308-1KT) levels in mice serum were detected by ELISA kit. In addition, ROS (#NG-EA691, ShangHaiYuanmin Biotechnology Co., Ltd.), TNF-α (R&D Systems, #DTA00D) and IL-6 (Sigma, #RAB0306-1KT) levels in HRGECs cellular supernatant were detected by ELISA kit.

### Western blot

Kidney tissues and HRGECs were lysed in RIPA lysis buffer (Roche, Germany) and the total protein content was determined by the BCA kit (Beyotime, China). The primary antibodies used were as follows: Anti-METTL14 (1:1000; Abcam, #ab252562, USA), Anti-α-Klotho (1:1000; Abcam, #ab181373, USA) and Anti-β-actin (1:10,000; Proteintech Group, USA). After incubated with HRP-conjugated secondary antibodies, the protein bands were visualized using an ECL kit (Pierce, USA) and analyzed using Tanon-4500 Gel Imaging System (Tanon, China).

### Quantitative realtime PCR (qRT-PCR)

The total RNA of renal tissues and HRGECs were extracted with RNAiso Reagent (TaKaRa, China) and then retro-transcribed with M-MLV Reverse Transcriptase (TaKaRa, China). qRT-PCR was performed with METTL14, Klotho gene specific primers and SYBR Green PCR Master Mix (Applied Biosystems, USA). GAPDH was used as endogenous control.

### m6A RNA immunoprecipitation PCR (RIP-qPCR)

The m6A-RIP test was carried out by Magna MeRIP m6A Kit (Merck, Germany) according to the manufacturer’s instructions. Total RNA was extracted and fragmented by ultrasound. The RNA fragment was incubated with Magnetic beads bound by anti-M6A antibody. After washed with m6A Salt, the binding RNA was eluted and purified by the RNA purification kit (Qiagen, USA) for qRT-PCR detection. The relative fold enrichment was calculated using 2^−ΔΔCt^ methods.

### Statistical analysis

All statistical analyses were performed using the SPSS software (SPSS Inc., USA). Data were produced as mean ± SD. When the data were normally distributed, they were analysed by unpaired two-tailed Student’s t tests and multiple groups were analysed by one-way analysis of variance (ANOVA). When the data were not normally distributed, nonparametric tests were used. P-value < 0.05 was considered significant.

## Results

### METTL14 was highly expressed in DN patients and high glucose-induced HRGECs

**Fig. 1:**
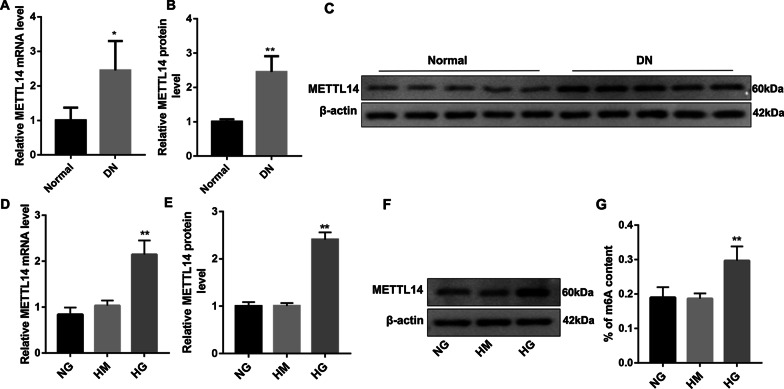
METTL14 was highly expressed in DN patients and high glucose-induced HRGECs. **A**–**C** Expression of METTL14 in kidney tissues of normal people and diabetic nephropathy patients was examined by qRT-PCR and western blot. Data are presented as the mean ± SD (n = 20); *P < 0.05, **P < 0.01 vs. normal. **D**–**F** Expression of METTL14 in glomerular endothelial cells was examined by qRT-PCR and western blot. **G** The m6A content was examined by colorimetric method. *NG* normal glucose, *HM* high mannitol, *HG* high glucose. Data are presented as the mean ± SD (n = 3); **P < 0.01 vs. NG

As shown in Fig. 1, METTL14 was significantly increased in kidney tissues of DN patients both at the mRNA and protein level (Fig. 1A–C). In high glucose (HG) induced HRGECs, the expression of METTL14 was also increased (Fig. 1D–F) compared with NG group. We also found that the m6A content was higher (Fig. 1G) in HG group examined by colorimetric method with m6A RNA methylation quantitative detection kit. These results indicated that METTL14 may be involved in the progress of diabetic nephropathy.

### METTL14 promoted high glucose-induced glomerular endothelial cell injury

**Fig. 2:**
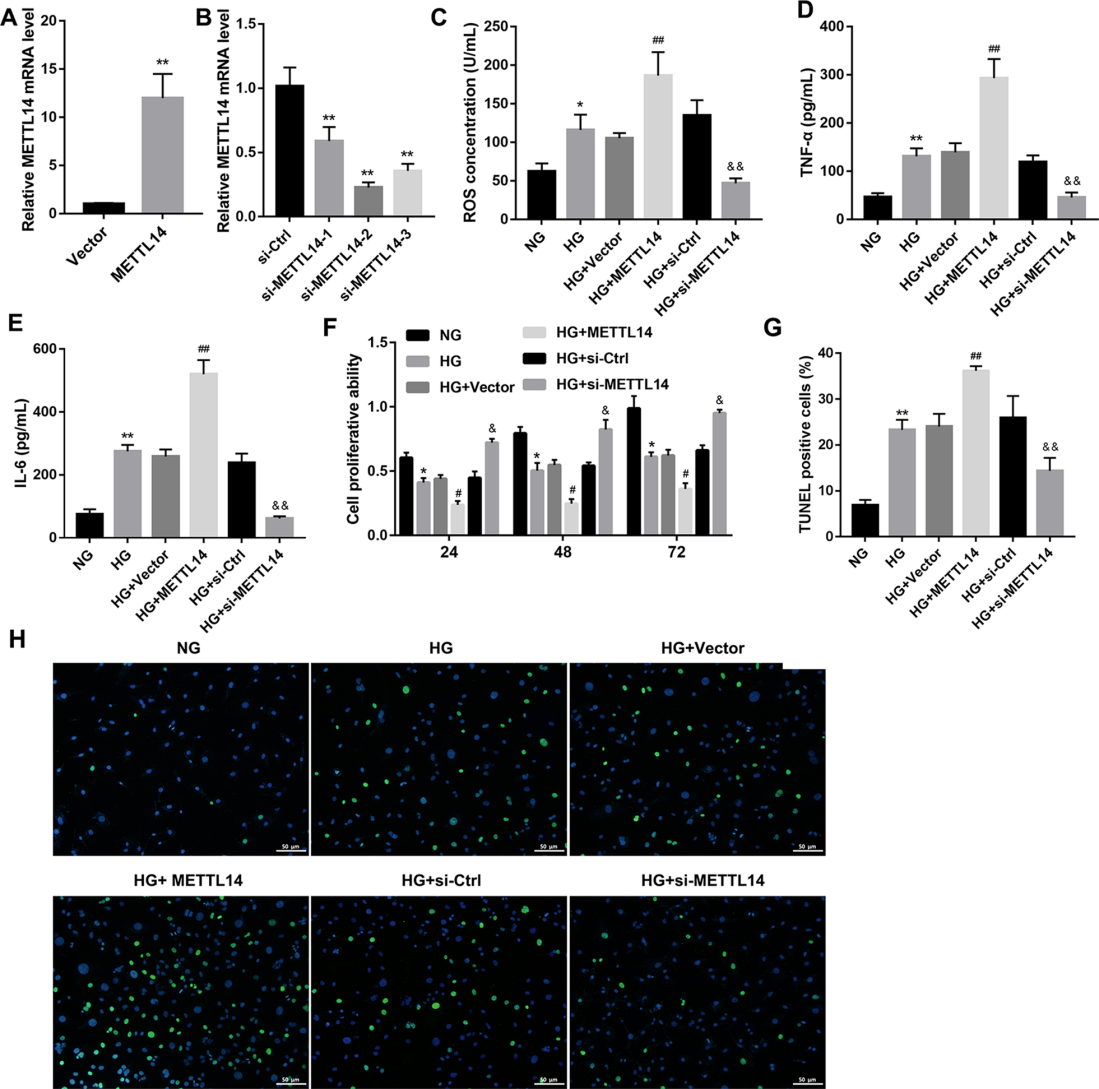
METTL14 promoted high glucose-induced glomerular endothelial cell injury. **A** Expression of METTL14 was examined by qRT-PCR after transfected with Vector or MELLT14 plasmid. **P < 0.01 vs. Vector. **B** Expression of METTL14 was examined by qRT-PCR after transfected with si-ctrl or MELLT14 siRNA. **P < 0.01 vs. si-Ctrl. **C**–**E** The levels of ROS, TNF-α and IL-6 were detected by ELISA. **F** Cell proliferation was assessed by CCK-8. **G**, **H** Cell apoptosis was analysed by TUNEL staining, Scale bar: 50 µm. *P < 0.05, **P < 0.01 vs. NG; ^#^P < 0.05, ^##^P < 0.01 vs. HG + Vector; ^&^P < 0.05, ^&&^P < 0.01 vs. HG + si-Ctrl. Data are presented as the mean ± SD (n = 3)

To explore the biological role of METTL14, Vector, METTL14 overexpression plasmid (METTL14), siRNA negative control (si-Ctrl) or METTL14 siRNA (si-METTL14-1, si-METTL14-2, si-METTL14-3) were transfected into HRGECs to overexpress or knockdown METTL14 (Fig. 2A, B). ELISA assay revealed (Fig. 2C–E) that the concentration of ROS, TNF-α and IL-6 were increased in high glucose-induced HRGECs and reached even higher levels after METTL14 overexpression, while decreased after knockdown of METTL14. In addition, overexpression of METTL14 markedly suppressed the cell proliferation (Fig. 2F) and promoted cell apoptosis (Fig. 2G, H) in high glucose-induced HRGECs. Conversely, METTL14 siRNA significantly promoted cell proliferation (Fig. 2F) but inhibited cell apoptosis (Fig. 2G, H). Together, these data suggested that METTL14 promoted high glucose-induced glomerular endothelial cell injury.

### METTL14 down-regulated α-klotho expression

**Fig. 3:**
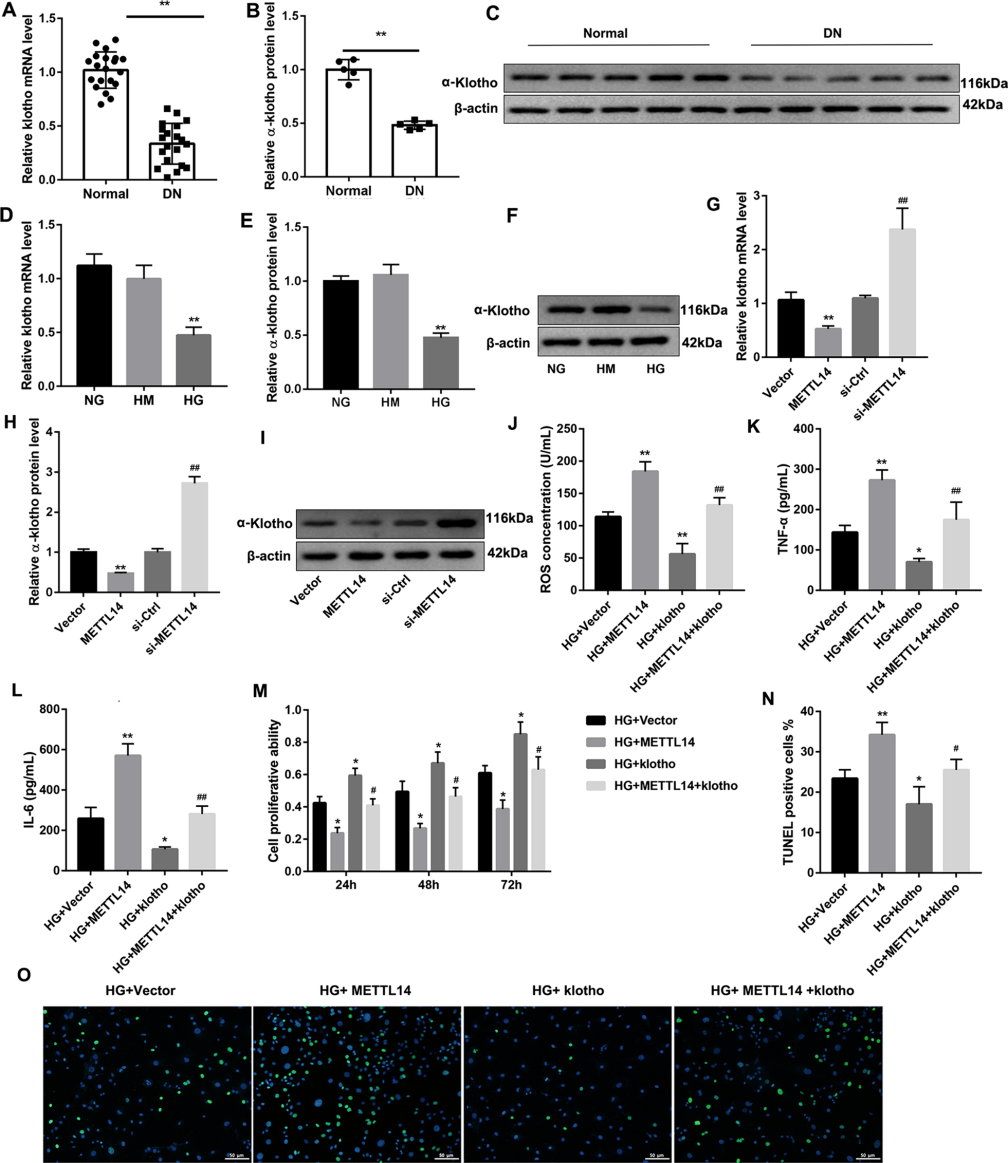
METTL14 down-regulated α-klotho expression. **A**–**C** Expression of α-klotho in kidney tissues of normal people and diabetic nephropathy patients was examined by qRT-PCR and western blot. Data are presented as the mean ± SD (n = 20); **P < 0.01 vs. Normal. **D**–**F** Expression of α-klotho in glomerular endothelial cells was examined by qRT-PCR and western blot. **P < 0.01 vs. HG. **G**–**I** Expression of α-klotho after overexpression or inhibition of METTL14. **P < 0.01 vs. Vector; ^##^P < 0.01 vs. si-Ctrl. **J**–**L** The levels of ROS, TNF-α and IL-6 were detected by ELISA. **M** Cell proliferation was assessed by CCK-8. **N**–**O** Cell apoptosis was analysed by TUNEL staining. *P < 0.05, **P < 0.01 vs. HG + Vector; ^#^P < 0.05, ^##^P < 0.01 vs. HG + METTL14. Data are presented as the mean ± SD (n = 3)

Consistent with our previous study (Wang et al. 2019), the mRNA and protein expression of α-klotho were down-regulated in DN patients and high glucose-induced HRGECs (Fig. 3A–F). Overexpression of METTL14 significantly decreased, but knockdown of METTL14 significantly increased the expression of α-klotho (Fig. 3G–I), indicating that METTL14 negatively regulated α-klotho. What’s more, increased ROS, TNF-α and IL-6 levels (Fig. 3J–L) and cell apoptosis (Fig. 3N, O), and decreased cell proliferation (Fig. 3M) in METTL14 overexpression cells were significantly rescued after co-transfection with Klotho expression plasmid. These findings suggested that α-klotho could be down-regulated by METTL14 and partially counteracted the function of METTL14 in glomerular endothelial cell injury.

### METTL14 regulated α-klotho m6A modification

**Fig. 4:**
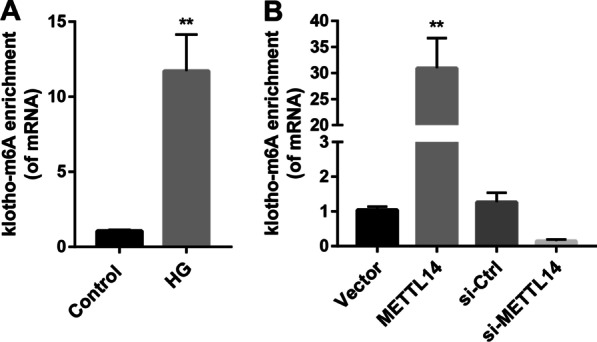
METTL14 regulated α-klotho m6A modification. **A** α-Klotho m6A level in high glucose induced cells was examined by RIP-qPCR. **P < 0.01 vs. Control. **B** α-Klotho m6A level after overexpression or inhibition of METTL14 was examined by RIP-qPCR. **P < 0.01 vs. Vector. Data are presented as the mean ± SD (n = 3)

As we know, METTL14 regulates gene expression through m6A modification. Here, we detected the m6A content of Klotho by RIP-qPCR. As displayed in Fig. 4A, the α-klotho m6A content was increased under high glucose condition. Overexpression of METTL14 increased the m6A level of α-klotho mRNA while METTL14 silence reduced the m6A level of α-klotho mRNA (Fig. 4B), indicating that METTL14 may mediate m6A modification of α-klotho.

### METTL14 aggravated renal injury and inflammation in db/db mice and rescued by Klotho

**Fig. 5:**
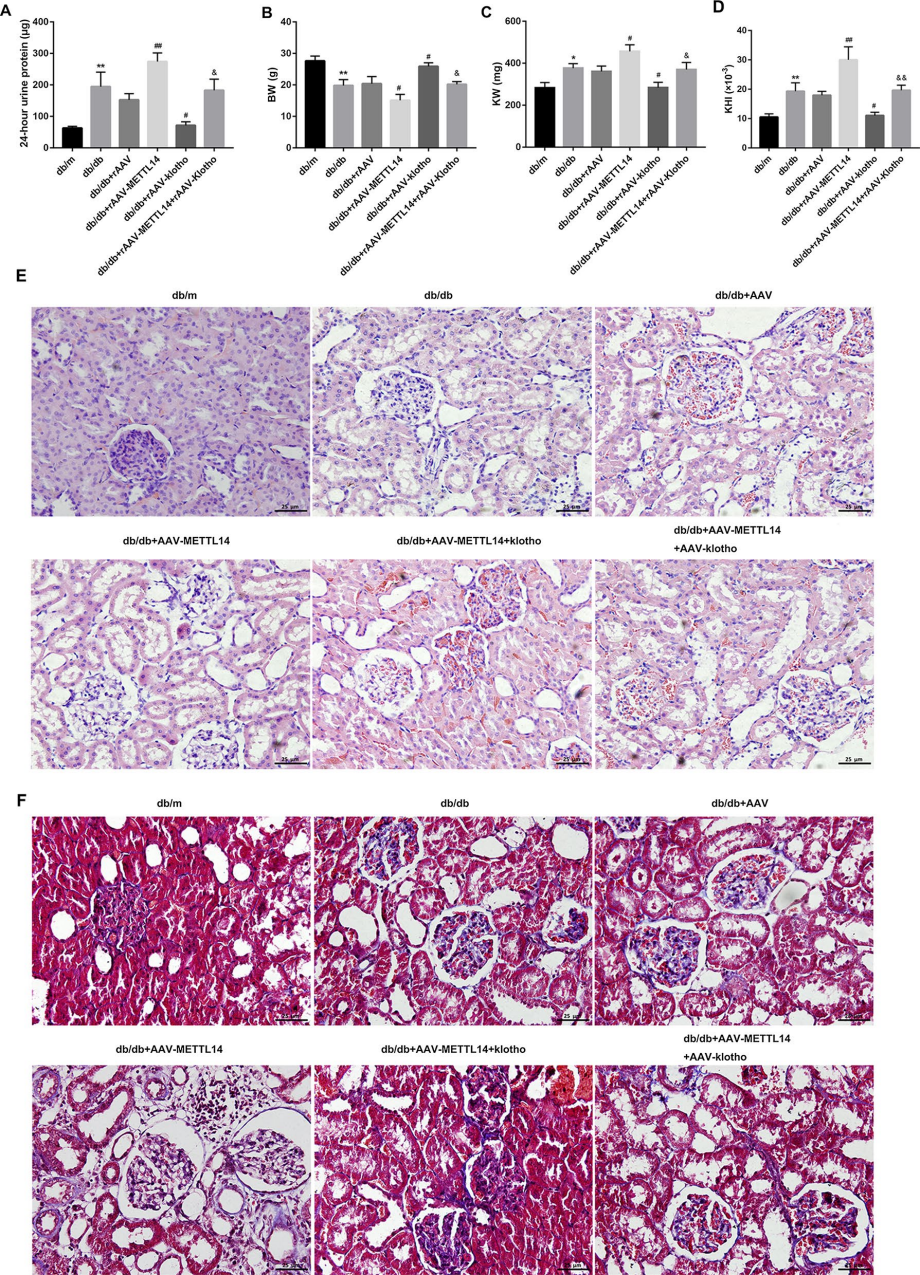
METTL14 promoted renal injury in db/db mice and rescued by α-klotho. **A**–**D** 24-h urine protein level, BW, KW and KHI was assessed. **E** H&E immunostaining was performed to detect general morphological changes. Scale bar = 25 µm. **F** Masson staining was assessed to examination of matrix deposition. Scale bar = 25 µm. *BW* body weight, *KW* kidney weight; KHI = KW/BW. Data are presented as the mean ± SD (n = 6); *P < 0.05, **P < 0.01 vs. db/m; ^#^P < 0.05, ^##^P < 0.01 vs. db/db + rAAV; ^&^P < 0.05, ^&&^P < 0.01 vs. db/db + rAAV-METTL14.

**Fig. 6:**
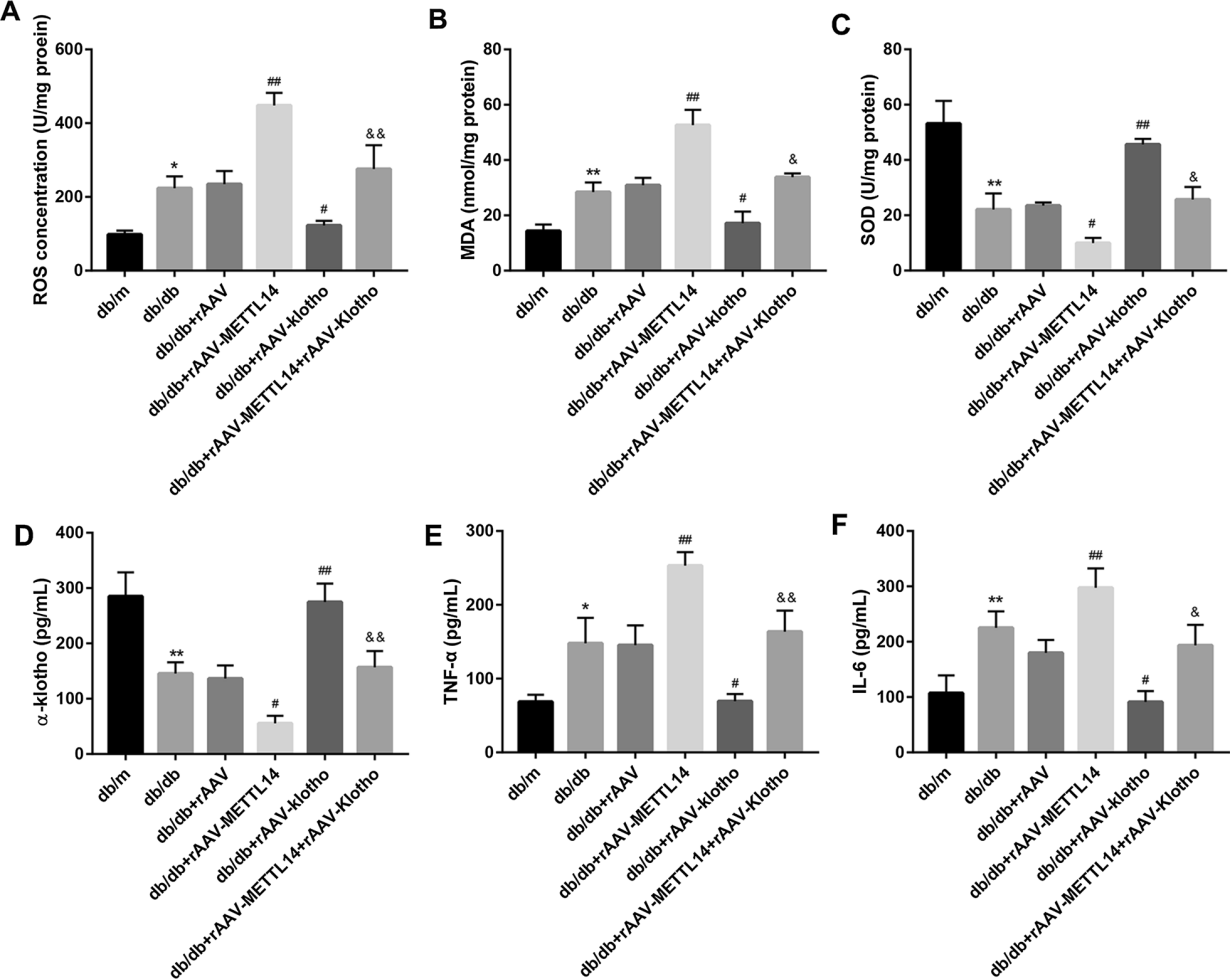
METTL14 promoted and α-klotho suppressed renal inflammation in db/db mice. **A**–**C** The levels of ROS, MDA, SOD in mice renal tissues, and **D**–**F** α-klotho, TNF-α and IL-6 in mice serum were detected by ELISA. Data are presented as the mean ± SD (n = 6); *P < 0.05, **P < 0.01 vs. db/m; ^#^P < 0.05, ^##^P < 0.01 vs. db/db + rAAV; ^&^P < 0.05, ^&&^P < 0.01 vs. db/db + rAAV-METTL14

To further confirm the role of METTL14 in vivo, db/db mice were treated with METTL14-expressed rAAV or Klotho-expressed rAAV. After administration of rAAV for 8 weeks, 24-h urine protein, kidney weight (KW), body weight (BW), renal injury were evaluated. As shown in Fig. 5A–D, rAAV-mediated overexpression of METTL14 significantly increased the levels of 24-h urine protein, KW and KHI but reduced the BW of db/db mice. By contrast, injection of rAAV-Klotho decreased the levels 24-h urine protein, KW and KHI but increased the BW of db/db mice, and even rescued the function of rAAV-METTL14. Moreover, H&E (Fig. 5E) and Masson staining (Fig. 5F) revealed that METTL14 overexpression exacerbated renal pathological alterations and collagen accumulation which could partially rescued by Klotho overexpression. The concentration of ROS, MDA in kidney tissues of db/db mice were notably increased and the levels of SOD was significantly decreased in db/db mice after injection of rAAV-METTL14. Overexpresssion of Klotho in contrast decreased the ROS and MDA but increased SOD levels in db/db mice or db/db + rAAV-METTL14 mice (Fig. 6A–C). In addition, the serum levels of TNF-α and IL-6 were increased while α-klotho level was decreased after injection of rAAV-METTL14, which were also reversed by rAAV-Klotho (Fig. 6D–F). Taking together, these results suggested that METTL14 aggravated renal injury of db/db mice which could be rescued by overexpression of α-klotho.

## Discussion

M6A modification has been proposed to be participates in many physiological and pathological processes. However, its roles in DN are unknown. In this work, we discovered the high content of m6A modification in DN, and identified the roles of METTL14 on glomerular endothelial cell injury in vitro and renal injury in vivo, uncovering the novel function of METTL14-mediated m6A modification in DN.

In the present study, we found that METTL14 is up-regulated in renal tissues of DN patients and high glucose-incubated glomerular endothelial cells. Overexpression of METTL14 promoted glomerular endothelial cells apoptosis and inflammation, and aggravated renal injury of DN mice. The roles of METTL14 in human diseases have been widely elucidated. For example, in cisplatin-induced acute kidney injury, overexpression of METTL14 promoted apoptosis of kidney proximal tubular cells (Zhou et al. 2019). In renal carcinoma, METTL14 was down-regulated and abrogated P2RX6 expression via m6A modification to suppress renal cancer cell migration and invasion (Gong et al. 2019). Xu et al. (2020) found that METTL14 knockdown protected the kidney against renal ischemic reperfusion injury. However, the mechanisms for METTL14 increased in DN and high glucose induced glomerular endothelial cells is unclear. Thus, more studies are needed in future.

Mechanistically, we have identified Klotho as a downstream target of METTL14 in DN. METTL14 overexpression significantly decreased α-klotho expression while silence of METTL14 significantly increased α-klotho expression. The m6A level of α-klotho mRNA was both increased under high glucose condition and after METTL14 overexpression, but reduced when METTL14 knockdown. Finally, overexpression of Klotho effectively abrogated the effect of METTL14 in glomerular endothelial cells and DN both in vitro and in vivo. These data demonstrated that the contribution of METTL14 in the DN progression has relied on α-klotho.

According to the literature, the expression of α-klotho in serum and urine klotho-to-creatinine ratio of patients with kidney disease is down regulated (Yi et al. 2020), such as renal fibrosis and podocyte injury (Cho et al. 2018), acute kidney injury (Qian et al. 2019), DN (Kacso et al. 2012), etc. α-klotho was found to be decreased during DN progress. Overexpression of α-klotho prevented renal injury of diabetic mice. It is reported that the expression of α-klotho is regulated by promoter methylation and histone deacetylation. For example, Masahiro et al. (2012) found that promoter methylation restricts klotho gene expression in renal tubular cells. In renal fibrosis, α-klotho was downregulated by histone deacetylation and restored by genistein through inhibiting histone 3 deacetylation of α-klotho promoter (Li et al. 2019b). Our study has shown that m6A RNA methylation contributed to the dysregulation of α-klotho.

As the development of m6A detecting techniques and the emerging of other novel technologies, a variety of m6A modification-related regulatory enzymes have been identified, facilitating the interpretation of their potential biological functions. M6A modification is regulated by methyltransferase (Writers), demethylase (Erasers) and binding proteins (Readers) (Yang et al. 2018). The main components of the methyltransferase complexes that have been discovered include METTL3, METTL14, WTAP and KIAA1429, while demethylases FTO and ALKBH5 are used as “Erasers” to remove the methylation. The biological function of m6A is mainly mediated by the “Reader” proteins through the selective recognition of m6A sites. The currently known “Reader” proteins include YTH domain proteins (YTHDF1, YTHDF2, YTHDF3, YTHDC1 and YTHDC2) and heterogeneous nuclear ribonucleoprotein HNRNP family proteins (HNRNPA2B1, HNRNPC and HNRNPG). METTL3 was up-regulated in patients with type 2 diabetes and mice with high-fat diet, and inhibited insulin sensitivity and promoted fatty acid metabolism (Xie et al. 2019). Proteomics analysis showed that high glucose induced high expression of WTAP in retinal pigmented epithelium cells (Chen et al. 2012). FTO has been reported to be associated with obesity and diabetes (Zhou et al. 2017; Mizuno 2018). Makiko et al. (2018) found that a variant in FTO was significantly associated with susceptibility to DN patients. YTHDC2 was found to be markedly down-regulated in obese mice and its overexpression improved the liver steatosis and insulin resistance through binding to the mRNA of lipogenic genes (Zhou et al. 2020). In addition, the whole exome sequencing has identified a variant of YTHDC2 which may contribute to type 2 diabetes susceptibility in Northeast India (Lalrohlui et al. 2020). Until now, the expression and roles of m6A methylation regulatory factors mentioned above in DN are unknown. Further studies will be required to research the functions of other regulatory factors to advance our understanding of m6A methylation in DN.

## Conclusions

In conclusion, our study found that METTL14 could aggravated high glucose-induced glomerular endothelial cell injury and diabetic nephropathy through m6A modification of α-klotho. The discovery of the METTL14-α-klotho pathway provides a new perspective for understanding of m6A modification in DN and is conducive to revealing new therapeutic targets.

## Data Availability

The datasets used and/or analysed during the current study are available from the corresponding author on reasonable request.

## References

[CR1] Azuma M, Koyama D, Kikuchi J, Yoshizawa H, Thasinas D, Shiizaki K, Kuro-o M, Furukawa Y, Kusano E (2012). Promoter methylation confers kidney-specific expression of the Klotho gene. FASEB J.

[CR2] Bi Z, Liu Y, Zhao Y, Yao Y, Wu R, Liu Q, Wang Y, Wang X (2019). A dynamic reversible RNA N(6)-methyladenosine modification: current status and perspectives. J Cell Physiol.

[CR3] Chen YH, Chen JY, Chen YW, Lin ST, Chan HL (2012). High glucose-induced proteome alterations in retinal pigmented epithelium cells and its possible relevance to diabetic retinopathy. Mol BioSyst.

[CR4] Chen J, Ning Y, Zhang H, Song N, Gu Y, Shi Y, Cai J, Ding X, Zhang X (2019). METTL14-dependent m6A regulates vascular calcification induced by indoxyl sulfate. Life Sci.

[CR5] Cho NJ, Han DJ, Lee JH, Jang SH, Kang JS, Gil HW, Park S, Lee EY (2018). Soluble klotho as a marker of renal fibrosis and podocyte injuries in human kidneys. PLoS ONE.

[CR6] Gao F, Wu H, Wang R, Guo Y, Zhang Z, Wang T, Zhang G, Liu C, Liu J (2019). MicroRNA-485-5p suppresses the proliferation, migration and invasion of small cell lung cancer cells by targeting flotillin-2. Bioengineered.

[CR7] Gong D, Zhang J, Chen Y, Xu Y, Ma J, Hu G, Huang Y, Zheng J, Zhai W, Xue W (2019). The m(6)A-suppressed P2RX6 activation promotes renal cancer cells migration and invasion through ATP-induced Ca(2+) influx modulating ERK1/2 phosphorylation and MMP9 signaling pathway. J Exp Clin Cancer Res.

[CR8] Han X, Sun Z (2020). Epigenetic regulation of KL (Klotho) via H3K27me3 (histone 3 lysine [K] 27 trimethylation) in renal tubule cells. Hypertension (dallas, Tex: 1979).

[CR9] Kacso IM, Bondor CI, Kacso G (2012). Soluble serum Klotho in diabetic nephropathy: relationship to VEGF-A. Clin Biochem.

[CR10] Kang WL, Xu GS (2016). Atrasentan increased the expression of klotho by mediating miR-199b-5p and prevented renal tubular injury in diabetic nephropathy. Sci Rep.

[CR11] Kuro OM (2019). The Klotho proteins in health and disease. Nat Rev Nephrol.

[CR12] Lalrohlui F, Zohmingthanga J, Hruaii V, Vanlallawma A, Senthil Kumar N (2020). Whole exome sequencing identifies the novel putative gene variants related with type 2 diabetes in Mizo population, northeast India. Gene.

[CR13] Li Y, Ren D, Xu G (2019). Long noncoding RNA MALAT1 mediates high glucose-induced glomerular endothelial cell injury by epigenetically inhibiting klotho via methyltransferase G9a. IUBMB Life.

[CR14] Li Y, Chen F, Wei A, Bi F, Zhu X, Yin S, Lin W, Cao W (2019). Klotho recovery by genistein via promoter histone acetylation and DNA demethylation mitigates renal fibrosis in mice. J Mol Med (berl).

[CR15] Liu J, Yue Y, Han D, Wang X, Fu Y, Zhang L, Jia G, Yu M, Lu Z, Deng X (2014). A METTL3-METTL14 complex mediates mammalian nuclear RNA N6-adenosine methylation. Nat Chem Biol.

[CR16] Liu J, Luo G, Sun J, Men L, Ye H, He C, Ren D (2019). METTL14 is essential for β-cell survival and insulin secretion. Biochim Biophys Acta.

[CR17] Liu S, Zhuo L, Wang J, Zhang Q, Li Q, Li G, Yan L, Jin T, Pan T, Sui X (2020). METTL3 plays multiple functions in biological processes. Am J Cancer Res.

[CR18] Mizuno TM (2018). Fat mass and obesity associated (FTO) gene and hepatic glucose and lipid metabolism. Nutrients.

[CR19] Qian Y, Che L, Yan Y, Lu R, Zhu M, Xue S, Ni Z, Gu L (2019). Urine klotho is a potential early biomarker for acute kidney injury and associated with poor renal outcome after cardiac surgery. BMC Nephrol.

[CR20] Rayego-Mateos S, Morgado-Pascual JL, Opazo-Ríos L, Guerrero-Hue M, García-Caballero C, Vázquez-Carballo C, Mas S, Sanz AB, Herencia C, Mezzano S (2020). Pathogenic pathways and therapeutic approaches targeting inflammation in diabetic nephropathy. Int J Mol Sci.

[CR21] Shen F, Huang W, Huang JT, Xiong J, Yang Y, Wu K, Jia GF, Chen J, Feng YQ, Yuan BF (2015). Decreased N(6)-methyladenosine in peripheral blood RNA from diabetic patients is associated with FTO expression rather than ALKBH5. J Clin Endocrinol Metab.

[CR22] Taira M, Imamura M, Takahashi A, Kamatani Y, Yamauchi T, Araki SI, Tanaka N, van Zuydam NR, Ahlqvist E, Toyoda M (2018). A variant within the FTO confers susceptibility to diabetic nephropathy in Japanese patients with type 2 diabetes. PLoS ONE.

[CR23] Vasanth Rao VR, Tan SH, Candasamy M, Bhattamisra SK (2019). Diabetic nephropathy: an update on pathogenesis and drug development. Diabetes Metab Syndr.

[CR24] Wang Q, Ren D, Li Y, Xu G (2019). Klotho attenuates diabetic nephropathy in db/db mice and ameliorates high glucose-induced injury of human renal glomerular endothelial cells. Cell Cycle (georgetown, Tex).

[CR25] Wang Y, Sun J, Lin Z, Zhang W, Wang S, Wang W, Wang Q, Ning G (2020). m(6)A mRNA methylation controls functional maturation in neonatal murine β-cells. Diabetes.

[CR26] Warren AM, Knudsen ST, Cooper ME (2019). Diabetic nephropathy: an insight into molecular mechanisms and emerging therapies. Expert Opin Ther Targets.

[CR27] Wei W, Ji X, Guo X, Ji S (2017). Regulatory role of N(6)-methyladenosine (m(6) A) methylation in RNA processing and human diseases. J Cell Biochem.

[CR28] Wu J, Frazier K, Zhang J, Gan Z, Wang T, Zhong X (2020). Emerging role of m(6) A RNA methylation in nutritional physiology and metabolism. Obes Rev.

[CR29] Xie W, Ma LL, Xu YQ, Wang BH, Li SM (2019). METTL3 inhibits hepatic insulin sensitivity via N6-methyladenosine modification of Fasn mRNA and promoting fatty acid metabolism. Biochem Biophys Res Commun.

[CR30] Xiong Y, Zhou L (2019). The signaling of cellular senescence in diabetic nephropathy. Oxid Med Cell Longev.

[CR31] Xu Y, Yuan XD, Wu JJ, Chen RY, Xia L, Zhang M, Han CH, Mou S (2020). The N6-methyladenosine mRNA methylase METTL14 promotes renal ischemic reperfusion injury via suppressing YAP1. J Cell Biochem.

[CR32] Yang Y, Hsu PJ, Chen YS, Yang YG (2018). Dynamic transcriptomic m(6)A decoration: writers, erasers, readers and functions in RNA metabolism. Cell Res.

[CR33] Yang Y, Shen F, Huang W, Qin S, Huang JT, Sergi C, Yuan BF, Liu SM (2019). Glucose is involved in the dynamic regulation of m6A in patients with type 2 diabetes. J Clin Endocrinol Metab.

[CR34] Yang J, Liu J, Zhao S, Tian F (2020). N(6)-methyladenosine mettl3 modulates the proliferation and apoptosis of lens epithelial cells in diabetic cataract. Mol Ther Nucleic Acids.

[CR35] Yi HJ, Lee JB, Lee KP, Oh YI, Song KH, Seo KW (2020). Investigation on urinary and serum alpha klotho in dogs with chronic kidney disease. BMC Vet Res.

[CR36] Zha X, Xi X, Fan X, Ma M, Zhang Y, Yang Y (2020). Overexpression of METTL3 attenuates high-glucose induced RPE cell pyroptosis by regulating miR-25-3p/PTEN/Akt signaling cascade through DGCR8. Aging.

[CR37] Zhou Y, Hambly BD, McLachlan CS (2017). FTO associations with obesity and telomere length. J Biomed Sci.

[CR38] Zhou P, Wu M, Ye C, Xu Q, Wang L (2019). Meclofenamic acid promotes cisplatin-induced acute kidney injury by inhibiting fat mass and obesity-associated protein-mediated m(6)A abrogation in RNA. J Biol Chem.

[CR39] Zhou B, Liu C, Xu L, Yuan Y, Zhao J, Zhao W, Chen Y, Qiu J, Meng M, Zheng Y (2020). N(6)-methyladenosine reader protein YT521-B homology domain-containing 2 suppresses liver steatosis by regulation of mRNA stability of lipogenic genes. Hepatology (baltimore, MD).

[CR40] Zhu Y, Cao X, Zhang X, Chen Q, Wen L, Wang P (2019). DNA methylation-mediated Klotho silencing is an independent prognostic biomarker of head and neck squamous carcinoma. Cancer Manage Res.

[CR41] Zou S, Toh JD, Wong KH, Gao YG, Hong W, Woon EC (2016). N(6)-Methyladenosine: a conformational marker that regulates the substrate specificity of human demethylases FTO and ALKBH5. Sci Rep.

