# Correction to: METTL14 promotes glomerular endothelial cell injury and diabetic nephropathy via m6A modification of α‑klotho

**DOI:** 10.1186/s10020-022-00437-0

**Published:** 2022-01-24

**Authors:** Manna Li, Le Deng, Gaosi Xu

**Affiliations:** Department of Nephrology, The Second Affiliated Hospital to Nanchang University, No. 1, Minde Road, Donghu District, Nanchang, 330006 China

## Correction to: Mol Med (2021) 27:106 10.1186/s10020-021-00365-5

Following publication of the original article (Li et al. 2021), the authors identified an error in Fig. 2. The correct Fig. 2 is given in this erratum.

**Fig. 2:**
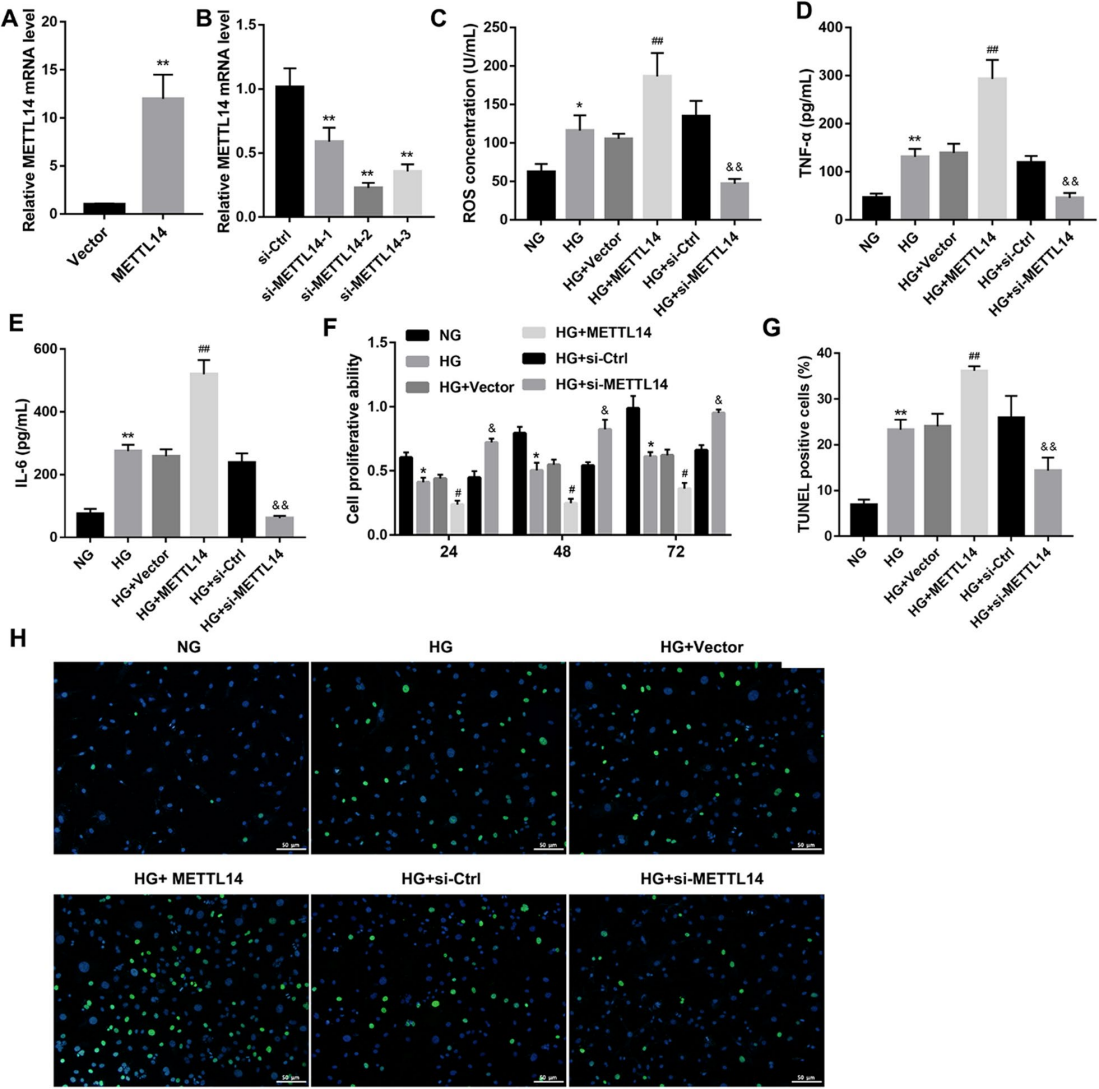
METTL14 promoted high glucose-induced glomerular endothelial cell injury. **A** Expression of METTL14 was examined by qRT-PCR after transfected with Vector or MELLT14 plasmid. **P < 0.01 vs. Vector. **B** Expression of METTL14 was examined by qRT-PCR after transfected with si-ctrl or MELLT14 siRNA. **P < 0.01 vs. si-Ctrl. **C**–**E** The levels of ROS, TNF-α and IL-6 were detected by ELISA. **F** Cell proliferation was assessed by CCK-8. **G**, **H** Cell apoptosis was analysed by TUNEL staining, Scale bar: 50 μm. *P < 0.05, **P < 0.01 vs. NG; ^#^P < 0.05, ^##^P < 0.01 vs. HG + Vector; ^&^P < 0.05, ^&&^P < 0.01 vs. HG + si-Ctrl. Data are presented as the mean ± SD (n = 3)

The original article has been corrected.
